# Tryptophan metabolic profile in term and preterm breast milk: implications for health

**DOI:** 10.1017/jns.2017.69

**Published:** 2018-04-04

**Authors:** Louise O'Rourke, Gerard Clarke, Aoife Nolan, Claire Watkins, Timothy G. Dinan, Catherine Stanton, R. Paul Ross, Cornelius Anthony Ryan

**Affiliations:** 1 Graduate Entry Medical School, University of Limerick, Limerick, Republic of Ireland; 2 Department of Psychiatry and Neurobehavioural Science, University College Cork, Cork, Republic of Ireland; 3 APC Microbiome Ireland, University College Cork, Cork, Republic of Ireland; 4 Teagasc, Food Research Centre, Moorepark, Fermoy, Co. Cork, Republic of Ireland; 5 College of Science, Engineering and Food Science, University College Cork, Cork, Republic of Ireland; 6 Department of Neonatology, Cork University Maternity Hospital, Cork, Republic of Ireland; 7 Department of Paediatrics and Child Health, University College Cork, Cork, Republic of Ireland

**Keywords:** Human milk, Tryptophan, Kynurenine, Kynurenic acid, Cytokines, Cortisol, EBM, expressed breast milk, IDO, indoleamine 2,3-dioxygenase, IFN-γ, interferon-γ, KynA, kynurenic acid, Kyn, kynurenine, MSD, Meso Scale Discovery, QC, quality control, TDO, tryptophan 2,3-dioxygenase, TRP, tryptophan

## Abstract

Breast milk is the only source of the essential amino acid tryptophan (TRP) in breast-fed infants. Low levels of TRP could have implications for infant neurodevelopment. The objectives of the present study were to compare the relationship of TRP and its neuroactive pathway metabolites kynurenine (Kyn) and kynurenic acid (KynA) in preterm and term expressed breast milk (EBM) in the first 14 d following birth, and the relationship of TRP metabolism to maternal stress and immune status. A total of twenty-four mothers were recruited from Cork University Maternity Hospital: twelve term (>38 weeks) and twelve preterm (<35 weeks). EBM samples were collected on days 7 and 14. Free TRP, Kyn and KynA were measured using HPLC, total TRP using MS, cytokines using the Meso Scale Discovery (MSD) assay system, and cortisol using a cortisol ELISA kit. Although total TRP was higher in preterm EBM in comparison with term EBM (*P* < 0·05), free TRP levels were lower (*P* < 0·05). Kyn, KynA and the Kyn:TRP ratio increased significantly in term EBM from day 7 to day 14 (*P* < 0·05), but not in preterm EBM. TNF-α, IL-6 and IL-8 were higher in day 7 preterm and term EBM in comparison with day 14. There were no significant differences between term and preterm EBM cortisol levels. Increased availability of total TRP, lower levels of free TRP and alterations in the temporal dynamics of TRP metabolism in preterm compared with term EBM, coupled with higher EBM inflammatory markers on day 7, may have implications for the neurological development of exclusively breast-fed preterm infants.

Breast milk is an excellent source of nutrition for growing infants due to its numerous bioactive factors such as cytokines, growth factors and hormones^(^^1^^)^. Breast milk continues an infant's exposure to the mother's immune system after birth, garnering passive immunity^(^^2^^,^^3^^)^, and playing a primary role in nurturing and regulating the immature infant immune system^(^^1^^,^^2^^)^. Tryptophan (TRP), present in breast milk, is an essential amino acid that can only be acquired through diet in humans^(^^4^^)^, and is one of the twenty amino acid building blocks required for protein synthesis^(^^5^^)^ in all cells in the body. Most (99 %) of TRP not used for protein synthesis is metabolised via the kynurenine (Kyn) pathway^(^^6^^)^. Two enzymes, tryptophan 2,3-dioxygenase (TDO) and indoleamine 2,3-dioxygenase (IDO), are involved in the Kyn pathway of TRP metabolism. TDO is primarily a stress-responsive enzyme^(^^5^^)^, while IDO is primarily an immune responsive enzyme^(^^7^^–^^9^^)^. Both the TDO and IDO pathways lead to the production of catabolites Kyn, neuroprotective kynurenic acid (KynA), and neurotoxic 3-hydroxykynurenine and quinolinic acid^(^^7^^)^.

Maternal serum levels of TRP fluctuate quite considerably, with the activity of TDO and IDO being tightly regulated both during and after pregnancy^(^^10^^)^. It has been established that IDO is necessary for the maintenance of pregnancy as it protects the fetus from T-cell-driven local inflammatory responses^(^^8^^,^^10^^)^. A study by Schröcksnadel *et al.*^(^^10^^)^ identified that maternal serum TRP levels progressively decreased during pregnancy via immune activation-induced IDO breakdown. In the postpartum period, though TRP began to rise again, TRP breakdown in this time period was more primarily triggered by activation of stress pathways. Of particular note, in the early peurperium, alterations in TRP catabolites are implicated in the development of affective symptoms^(^^11^^)^.

Cortisol, which is present in breast milk, is a glucocorticoid that is released under the control of the hypothalamic–pituitary–adrenal axis in a diurnal pattern, with highest levels in the morning and lowest levels in the evening^(^^12^^)^, and is integral to the correct development of the central nervous system^(^^13^^)^. While antenatal glucocorticoids are vital for normal development of the fetus^(^^14^^)^, excess secretion due to maternal stress in pregnancy are strongly associated with preterm birth^(^^15^^)^, and with infant behaviour, mood, cognition and attention abnormalities in later life^(^^16^^,^^17^^)^. In the postnatal period, higher levels of maternal cortisol are associated with fearfulness in breastfed infants^(^^13^^)^. Aside from TRP's protein synthesis function, TRP is also the primary precursor for serotonin and melatonin^(^^18^^)^, needed for the regulation of immune responses, behaviour, mood, appetite, haemodynamics and growth^(^^5^^)^. Cortisol activates the TDO-led breakdown of TRP, leading directly to a decrease in brain serotonin synthesis^(^^7^^)^. Deficiencies in serotonin levels are seen as significant causative factors in the development of anxiety, aggression, affective disorders and stress syndromes^(^^5^^)^. Furthermore, melatonin, the by-product of serotonin, also found in breast milk, plays a crucial role in stabilising the circadian rhythm of the newborn and consolidation of the sleep–wake cycle, for optimal development of the brain, and in immune defence^(^^7^^,^^19^^)^. Therefore, alterations in TRP have the potential for neurodevelopmental consequences^(^^7^^,^^20^^)^.

The relationship dynamic between the serotonin precursor TRP, its neuroactive pathway metabolites (Kyn and KynA), immunity and stress has not to our knowledge been previously investigated in term and preterm expressed breast milk (EBM). In this study, we hypothesised that higher stress levels associated with preterm birth would be reflected in preterm EBM, resulting in increased levels of cortisol and decreased levels of TRP in comparison with term EBM. As immune activation is also associated with preterm labour^(^^21^^)^, and as cytokines activate IDO-led breakdown of TRP down the Kyn metabolite pathway, we also hypothesised that we would find higher levels of immune activation in preterm EBM.

The aims of the study are twofold: (a) compare the relationship of serotonin precursor TRP and its neuroactive pathway metabolites Kyn and KynA, in preterm and term EBM in the first 14 d following birth; (b) examine the relationship of TRP to maternal stress (EBM cortisol) and immune status (EBM cytokines – interferon-γ (IFN-γ), TNF-α, IL-1, IL-6, IL-8) over the same time-frame.

## Methods

This study was conducted in the neonatology department of the Cork University Maternity Hospital over an 8-week period from June to July 2013. All mothers received verbal and written information about the study and signed a written consent form before participation. Demographic data were collected on each mother and baby.

Two specimens of EBM (hind milk) (5–10 ml) were collected from all mothers post-partum, on day 7 and on day 14 from their homes. Every care was taken to ensure that the timing and collection of the EBM samples were carefully controlled and consistent. There was only one collecting investigator who spoke with all mothers either via telephone or in person the evening before each time point on day 6 and day 13 to ensure they fully understood the process. All samples were manually expressed after the first feed of the day, typically between 06.00 and 08.00 hours. Of the mothers who provided EBM samples from home, once the hind-milk sample was manually expressed into sterile polypropylene containers, they were instructed to keep the sample in their fridge until collection by the collecting researcher the same day no later than 14.00 hours. Any mothers and infants still on the neonatal unit used the neonatal sample collection fridge for storage. During transport, the samples were stored in a cooler on packed ice (±4°C). Limited transportation times were ensured by placing a 25-mile (40-km) radius restriction for participation in the study. At the laboratory, all samples were stored initially at −20°C and then at −80°C until assayed.

The exclusion criteria included maternal use of antibiotics, maternal mastitis, and mothers living further than a 25-mile (40-km) radius from the hospital. Five mothers who were initially recruited onto the study had to be excluded (four mothers were commenced on antibiotics and one mother had difficulties with breastfeeding). The study was granted ethics approval by the Clinical Research Ethics Committee of the Cork Teaching Hospitals (reference number of approval: ECM (IIII) 06/08/13).

Free TRP (TRP bound to/dissociated from albumin) and its Kyn pathway metabolites were determined using HPLC coupled to fluorescent and UV detection as previously described^(^^22^^)^. Briefly, EBM samples were spiked with an internal standard (3-nitro l-tyrosine) prior to the addition of 20 µl of 4 m-perchloric acid to 200 µl of sample. Samples were centrifuged at 21 000 ***g*** on a Hettich Mikro 22R centrifuge (AGB) for 20 min at 4°C and 100 µl of supernatant fraction transferred to an HPLC vial for analysis on the HPLC system (UV and fluorescence detector detection). All samples were injected onto a reversed-phase Luna 3 µm C18 (2) 150 × 2mm column (Phenomenex), which was protected by Krudkatcher disposable pre-column filters (Phenomenex) and Security Guard cartridges (Phenomenex). The mobile phase consisted of 50 mm-acetic acid, 100 mm-zinc acetate with 3 % (v/v) acetonitrile and was filtered through Millipore 0·45 µm HV Durapore membrane filters (AGB) and vacuum degassed prior to use. Compounds were eluted isocratically over a 30-min runtime at a flow rate of 0·3 ml/min after a 20 µl injection. The column was maintained at a temperature of 30°C and samples/standards were kept at 8°C in the cooled autoinjector prior to injection. The fluorescent detector was set at an excitation wavelength of 254 nm and an emission wavelength of 404 nm. The UV detector was set to 330 nm. TRP and its metabolites (Kyn, KynA) were identified by their characteristic retention times as determined by standard injections that were run at regular intervals during the sample analysis. Analyte:internal standard peak height ratios were measured and compared with standard injections, and results were expressed as ng of analyte per ml of EBM.

Total TRP (TRP content of EBM protein) in EBM was determined using GC-MS, which is a modified version of the protocol described by Smart *et al.*^(^^23^^)^. A pooled quality control (QC) sample was created by collecting a small aliquot from each of the samples. Prior to analysis, all samples were hydrolysed by the addition of 4 mm-sodium hydroxide followed by 10 h at 121°C and 5-methyltryptophan was added as an internal standard. QC samples were hydrolysed individually. All samples were then derivatised with methyl chloroformate. The samples were randomised and analysed by GC-MS. The QC sample was analysed every four to six samples. Testing of matrix effects was performed by spiking/dilution of QC samples. It is assumed that the individual samples do not have matrix effects not found in the QC samples.

A Cortisol ELISA kit (Enzo Life Sciences) was used to determine the concentration of cortisol in EBM. This technique has been described for use in human breast milk by Silva *et al.*^(^^24^^)^. Samples were thawed on ice and centrifuged at 2000 ***g*** for 10 min at 4°C to de-fat the EBM. The aqueous phase was extracted and used in the ninety-six-well-plate ELISA assay. Samples were incubated with a solution of alkaline phosphatase conjugated with cortisol along with a monoclonal antibody for cortisol for 2 h at room temperature. The plate was washed three times in wash solution and incubated with *p*-nitrophenyl phosphate substrate solution for 1 h. Stop solution was applied to each well and the optical density was read immediately at 405 nm. All samples were run in duplicate, blanked against the blank wells and their concentration determined against kit standards. Results are expressed as pg per ml of sample.

For cytokine analysis, a Meso Scale Discovery (MSD) assay system was used following a technique previously described for use in human breast milk by Semba *et al.*^(^^25^^)^. Milk was prepared as described for cortisol assays. Samples were centrifuged at 2000 ***g*** for 10 min at 4°C and the aqueous layer aspirated. A Human Pro-inflammatory II 4-Plex Ultra-Sensitive Kit (MSD) was used to determine IL-1β, IL-6, IL-8 and TNF-α concentrations in the EBM samples. A Single Spot Human Cytokine Assay System (MSD) for IFN-γ was also run. Samples and standards were run in duplicate and the concentration was measured using the MSD Discovery Workbench® analysis software. Results are presented in pg per ml of sample.

Statistical analysis was performed using the Graphpad Prism software package. Data were analysed using two-way repeated-measures ANOVA (matched pairs only) and two-way ANOVA (all data points, missing values not replaced) followed by *post hoc* analysis (Bonferroni multiple comparisons) as appropriate. *P* < 0·05 was considered significant. As our preliminary dataset was not suitable for data imputation, we provided an analysis restricted to individuals with a complete dataset and also analysed all data points available to maintain the power of the analysis and to give a more complete picture of the data^(^^26^^)^. See Supplementary Table S1 for the number of data points used.

## Results

A total of twenty-nine lactating mothers were recruited, of whom twenty-four successfully completed the study: twelve mothers with term babies (>38 weeks; term group), and twelve mothers with preterm babies (<35 weeks; preterm group). The demographics of mothers and infants, term and preterm, are presented in Table 1.

**Table 1.** Demographics of mothers and infants (Mean values, standard deviations and ranges; percentages)

	Term group (*n* 12)			Preterm group (*n* 12)		
	Mean	sd	Range	Mean	sd	Range
Maternal age (years)	34·4	3·75	29–44	37·0	7·16	29–44
Parity	1·92	0·9	1–3	1·71	0·5	1–4
Delivery (%)						
SVD	67			25		
CS	33			75		
Gestational age (weeks)	39·7	0·9	38·1–41·7	32·6	1·8	29·0–34·4
Birth weight (kg)	3·5	0·6	2·6–4·3	2·0	0·4	1·4–2·8

SVD, spontaneous vaginal delivery; CS, Caesarean section.

### Expressed breast milk total and free tryptophan

**Fig. 1.**
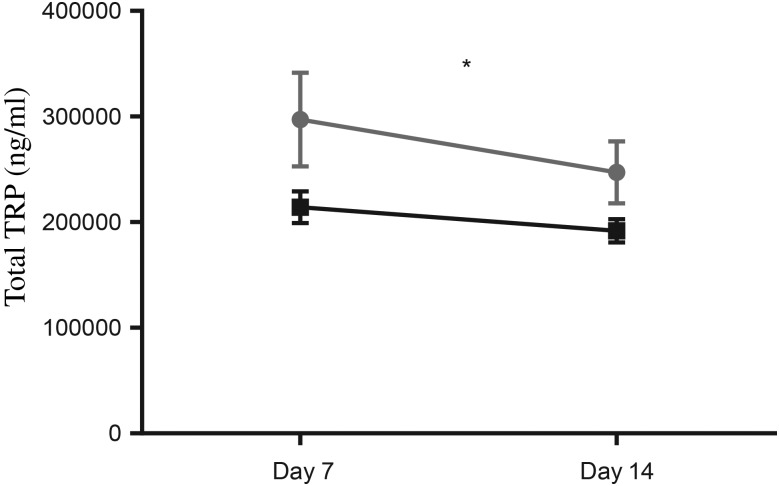
Total tryptophan (TRP) in term (-■-) and preterm (-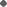-) expressed breast milk (EBM) from day 7 to day 14. Results are means, with standard errors represented by vertical bars. See Supplementary Table S1 for the number of data points used. Total TRP was significantly higher in preterm EBM compared with term EBM (two-way ANOVA; *P* < 0·05). * *P* < 0·05 (two-way ANOVA).

Total TRP was significantly higher in preterm EBM, compared with term EBM (two-way ANOVA, *P* < 0·05; Fig. 1). There were no significant differences observed between day 7 and day 14 samples in preterm or term EBM (*P* = 0·24 and 0·42, respectively).

**Fig. 2.**
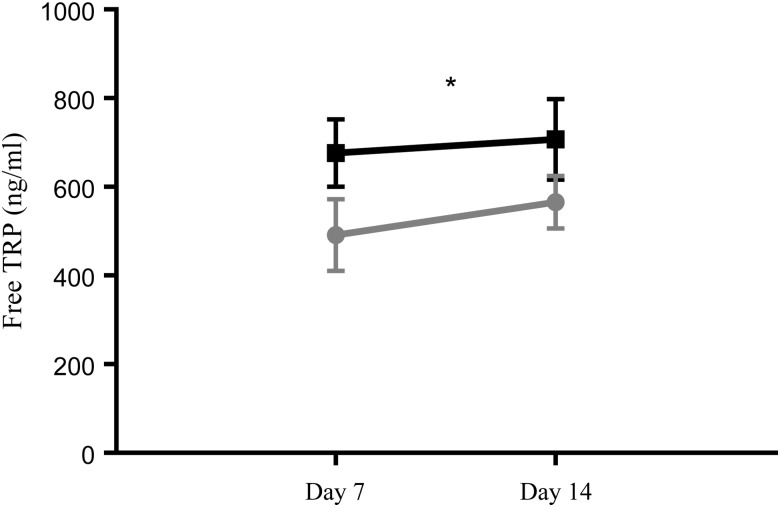
Free tryptophan (TRP) levels in term (-■-) and preterm (-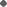-) expressed breast milk (EBM) from day 7 to day 14. Results are means, with standard errors represented by vertical bars. See Supplementary Table S1 for the number of data points used. Free TRP levels were significantly lower in preterm EBM compared with term EBM (*P* < 0·05). * *P* < 0·05 (two-way ANOVA).

In contrast to total TRP, free TRP levels were significantly lower in preterm EBM compared with term EBM (two-way ANOVA, *P* < 0·05; Fig. 2). There was no effect of time on free TRP concentrations (*P* > 0·05), nor was there an interaction between time and group. A *post hoc* analysis did not indicate any differences between groups (*P* > 0·05). A matched-pairs analysis indicated no significant effect of group, time, or interaction between group and time (*P* > 0·05).

### Expressed breast milk kynurenine

**Fig. 3.**
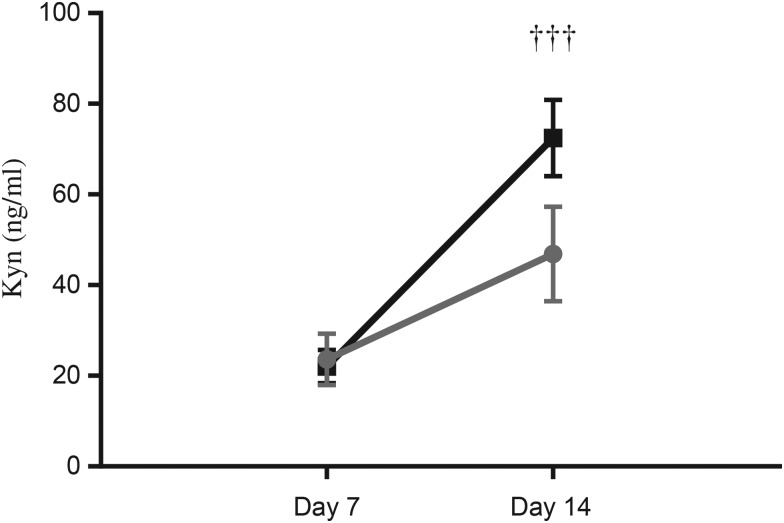
Kynurenine (Kyn) levels in term (-■-) and preterm (-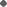-) expressed breast milk (EBM) on day 7 and day 14. Results are means, with standard errors represented by vertical bars. See Supplementary Table S1 for the number of data points used. Kyn levels increased significantly in term EBM from day 7 to day 14 (*P* < 0·01); the increase was not significant in preterm EBM (*P* > 0·05). ††† *P* < 0·001 (two-way ANOVA).

There were no significant differences in Kyn levels comparing term and preterm EBM. However, while mean Kyn levels on day 14 were higher compared with day 7 levels in term EBM (72·5 se 8·4) *v.* 22·1 (se 3·7) ng/ml, two-way ANOVA, *P* < 0·001; Fig. 3), the differences were not significant in preterm EBM (23·6 (se 5·7) *v.* 46·9 (se 10·5) ng/ml; *P* > 0·05). While Kyn levels in term EBM indicated an effect of time (*P* < 0·001), no effect of group and no interaction between group and time were seen (*P* > 0·05, all data points). A matched-pairs analysis of Kyn levels in term EBM also highlighted an effect of time (*P* < 0·001).

### Expressed breast milk kynurenic acid

**Fig. 4.**
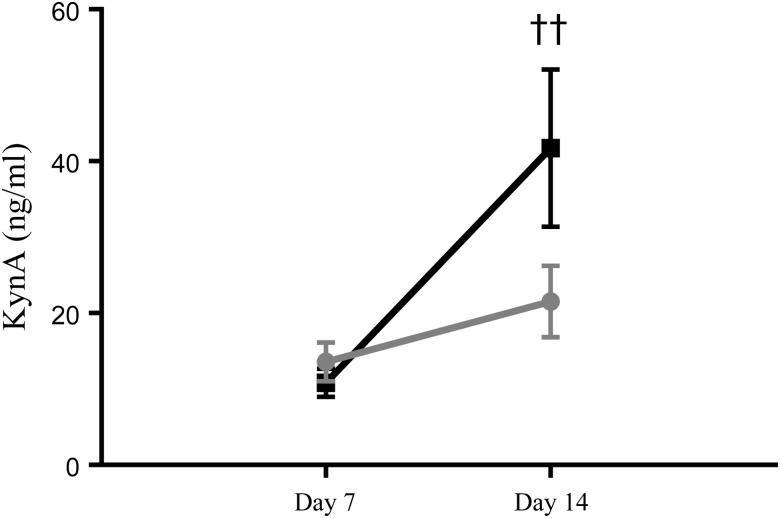
Kynurenic acid (KynA) levels in term (-■-) and preterm (-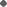-) expressed breast milk (EBM) on day 7 and day 14. Results are means, with standard errors represented by vertical bars. See Supplementary Table S1 for the number of data points used. KynA levels increased significantly in term EBM from day 7 to day 14 (*P* < 0·01); the increase was not significant in preterm EBM (*P* > 0·05). †† *P* < 0·01 (two-way ANOVA).

KynA levels in term EBM indicated an effect of time (*P* < 0·01) but no effect of group and no interaction between group and time (*P* > 0·05, all data points). A matched-pairs analysis of KynA levels in term EBM also highlighted an effect of time (*P* < 0·01). A *post hoc* analysis indicated that KynA levels increased significantly in term EBM from day 7 to day 14 (10·8 (se 1·8) *v.* 41·7 (se 10·3) ng/ml, two-way ANOVA, *P* < 0·01; Fig. 4). The KynA level increase was not significant in preterm EBM from day 7 to day 14 (13·6 (se 2·6)  *v.* 21·5 (se 4·7) ng/ml; *P* > 0·05).

### Expressed breast milk kynurenine:tryptophan ratio

**Fig. 5.**
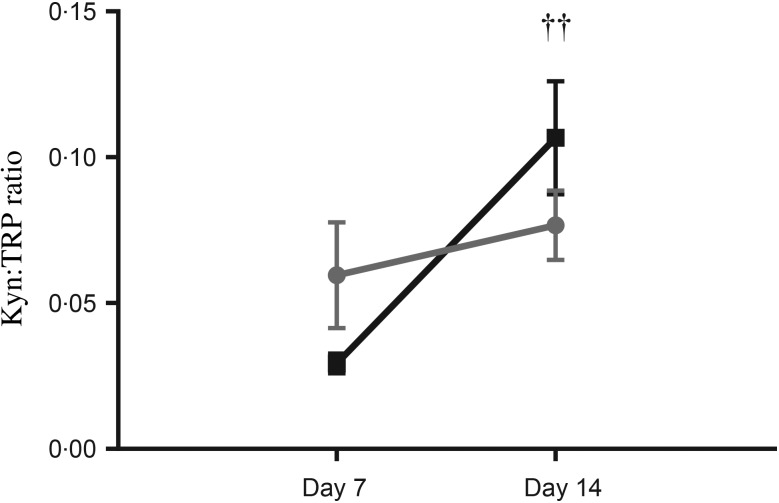
Kynurenine:tryptophan (Kyn:TRP) ratio in term (-■-) and preterm (-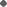-) expressed breast milk (EBM) on day 7 and day 14. Results are means, with standard errors represented by vertical bars. See Supplementary Table S1 for the number of data points used. The Kyn:TRP ratio increased significantly from day 7 to day 14 in term EBM (*P* < 0·01); the increase was not significant in preterm EBM (*P* > 0·05). †† *P* < 0·01 (two-way ANOVA).

An indicator of TRP breakdown, the Kyn:TRP ratio showed an effect of time (*P* < 0·01, all data points) but no effect of group and no interaction between group and time. A matched-pairs analysis of the Kyn:TRP ratio in term EBM indicated a trend towards an effect of time (*P* = 0·05). A *post hoc* analysis indicated that the Kyn:TRP ratio increased significantly from day 7 to day 14 in term EBM (0·029 (se 0·003)  *v.* 0·11 (se 0·02) ng/ml, two-way ANOVA, *P* < 0·01; Fig. 5). The Kyn:TRP ratio increase was not significant in preterm EBM (0·06 (se 0·02)  *v.* 0·08 (se 0·01) ng/ml; *P* > 0·05).

### Expressed breast milk free tryptophan:total tryptophan ratio

**Fig. 6.**
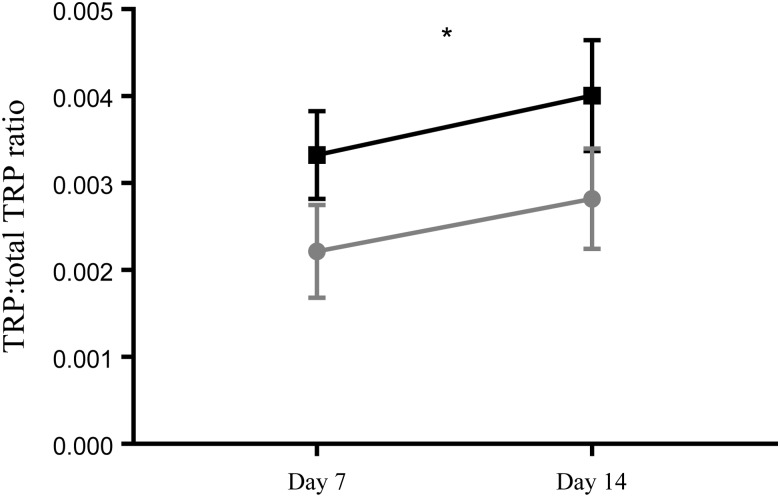
Free tryptophan (TRP):total TRP ratio in term (-■-) and preterm (-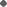-) expressed breast milk (EBM) on day 7 and day 14. Results are means, with standard errors represented by vertical bars. See Supplementary Table S1 for the number of data points used. There was an overall significant difference between the term and preterm group (*P* < 0·05). The free TRP:total TRP ratio was significantly lower in the preterm EBM (*P* < 0·05) compared with term EBM. * *P* < 0·05 (two-way ANOVA).

There was no effect of time, nor was there an interaction between time and group on the free TRP:total TRP ratio. There was an overall significant difference between the term and preterm group (two-way ANOVA, *P* < 0·05; Fig. 6). The free TRP:total TRP ratio was significantly lower in the preterm EBM (*P* < 0·05) compared with term EBM.

### Expressed breast milk inflammatory markers

There were significantly higher levels of TNF-α and IL-8, and a trend towards elevated IL-6 in both term and preterm EBM on day 7 compared with day 14 (*P* < 0·05; Table 2). There was no effect on group (*P* > 0·05) and no interaction effect (*P* > 0·05) on any of the inflammatory cytokines (Table 2). *Post hoc* analysis indicated that the time-dependent decrease in these cytokines was not significant in either group. There was no effect of group or time and no interaction between group and time for other EBM pro-inflammatory cytokines (Table 2).

**Table 2.** Pro-inflammatory cytokine levels (pg/ml) in term and preterm expressed breast milk (EBM) on day 7 and day 14, and *P* values for group (G), time (Ti) and interaction (I)† (Mean values and standard deviations)

		Day 7		Day 14		
	Group	Mean	sd	Mean	sd	*P*
Interferon-γ	Term	0·9	0·4	1·4	0·4	0·1 (G)
	Preterm	0·3	0·1	0·6	0·2	0·3 (Ti)
						0·8 (I)
TNF-α	Term	17·2	4·1	9·0	2·3	0·9 (G)
	Preterm	13·6	3·8	10·6	3·1	0·03* (Ti)
						0·7 (I)
IL-1β	Term	18·4	6·1	11·4	4·7	0·6 (G)
	Preterm	16·1	8·4	9·3	2·6	0·2 (Ti)
						0·8 (I)
IL-6	Term	22·2	5·9	9·1	2·9	0·9 (G)
	Preterm	20·6	7·6	9·4	3·5	0·06 (Ti)
						0·6 (I)
IL-8	Term	440·2	112·7	267·9	74·8	0·6 (G)
	Preterm	384·2	148·3	232·7	73·3	0·03* (Ti)
						1·0 (I)
Cortisol	Term	3624	572·5	2982	348·9	0·8 (G)
	Preterm	3221	1045·0	3793	1018·0	0·9 (Ti)
						0·4 (I)

* Significant differences noted.

† See Supplementary Table S1 for the number of data points used.

### Expressed breast milk cortisol

There were no differences between term and preterm EBM levels, with no effect of group or time and no interaction between group and time for either term or preterm EBM cortisol (Table 2).

## Discussion

In the present study, we investigated the supply dynamics of TRP and its Kyn pathway metabolites (Kyn and KynA), cortisol and cytokines, in paired EBM samples from mothers of term and preterm infants at two time points post-delivery. To our knowledge, this is the first demonstration showing that total TRP was found to be significantly higher in preterm EBM compared with term EBM, and preterm EBM contains less free TRP than term EBM. In addition, TRP metabolites, Kyn and KynA, have been identified and quantified in EBM. Notably, higher levels of these metabolites were found in term day 14 EBM compared with term day 7 EBM. Higher levels of inflammatory markers were identified in term and preterm EBM on day 7 compared with day 14.

We have noted a reduced availability of free TRP and increased total TRP concentrations in preterm EBM. This broad trend is mirrored in the literature where preterm milk in comparison with term milk is associated with higher protein content in the early stages of the lactation cycle^(^^27^^–^^32^^)^. The increase in total TRP in preterm EBM in the present study reflects the portion that requires processing via digestion but probably contributes more substantially to circulating TRP availability on a longer timescale. Of note, 95–99 % of amino acids in breast milk are contained within proteins^(^^33^^)^. Conversely, as free amino acids in breast milk are more readily absorbed than protein-derived amino acids, they may be a source of easily accessible N-containing compounds for the infant; they may have a role in protecting intestinal growth; and may serve as functional substrates to nervous tissues^(^^34^^)^.

Unlike other amino acids, 90–95 % of free TRP binds primarily to albumin in plasma or serum, while the remainder is used in its dissociated form for immediate uptake by tissues and organs^(^^35^^)^. Free TRP binding to albumin can be influenced by many factors such as hormones, nutrition, pregnancy, drugs and NEFA^(^^5^^,^^35^^)^. In this preliminary study, we chose to use hind milk primarily to avoid interfering with infant feeding^(^^34^^,^^36^^)^. Hind milk has a much higher fat content in comparison with aqueous-rich foremilk^(^^37^^–^^39^^)^, and, consequently, higher levels of NEFA which could have an effect on albumin-bound TRP. However, the dynamic involving albumin-bound TRP is more important for circulating TRP where albumin is present in much higher concentrations compared with the relatively lower albumin levels in breast milk where its physiological role is less well studied. Our analysis of free TRP in EBM does not distinguish between the proportion which is bound and unbound to albumin. This better captures the breast milk supply of TRP that is not a constituent of protein to the infant since it is unlikely that TRP would remain associated with albumin in the gastrointestinal tract once consumed.

In recognition of the importance of TRP in brain development, ESPGHAN coordinated international expert group global standard guidelines for the composition of infant formula^(^^40^^)^ recommend that human milk protein composition should be used as the reference when determining formula protein composition on the basis that this achieves optimal circulating TRP levels. There are many different kinds of commercial cows’ milk-based formulas available intended for different periods in life, adapted and follow-on, powdered or liquid^(^^41^^,^^42^^)^; full-term infant formulas where proteins can be intact, hydrolysed or synthetic; preterm formulas with higher energy density; and specialised formulas, for example extensively hydrolysed protein formulas or formulas with free amino acids for babies with cows’ milk insensitivity or absorption issues^(^^42^^)^. While human milk contains approximately 114 mg/g N content of TRP^(^^40^^)^, it has been found that many registered formulas have lower mean TRP concentrations than in breast milk (1–1·5 *v.* 2·5 %)^(^^43^^)^. Comparable circulating plasma TRP concentrations can be achieved following direct supplementation of formula with TRP^(^^44^^)^; however, the static nature of infant formula is unable to support the development of circadian rhythms comparable with breast milk^(^^45^^)^. Breast milk TRP demonstrates a nocturnal acrophase, peaking at 03.00 hours and at its lowest level at 15.00 hours in all three stages of lactation (colostrum, transitional and mature breast milk)^(^^45^^)^.

Breast milk is the only source of the essential amino acid TRP for exclusively breast-fed infants. TRP is vital for optimal growth^(^^45^^)^, and central nervous system development^(^^27^^)^, in early infancy, and at no other time is structural demand for TRP higher than in the neonatal period^(^^45^^)^. Brain serotonin synthesis is proportional to the amount of peripheral TRP available for transport into the brain; thus any decrease in peripheral TRP supply can potentially have an impact on brain serotonin levels^(^^4^^,^^5^^)^. An intricate and complex balance exists between TRP and its Kyn pathway metabolites^(^^7^^,^^20^^)^. Free TRP and Kyn readily cross the blood–brain barrier via competitive large amino acid transporters, while KynA, 3-hydroxykynurenine and quinolinic acid cross the blood–brain barrier at relatively low rates^(^^7^^,^^20^^,^^46^^)^. A high ratio of KynA:3-hydroxykynurenine and quinolinic acid is associated with low cognitive performance (e.g. schizophrenia), while a low ratio of KynA:3-hydroxykynurenine and quinolinic acid is associated with high neuronal vulnerability (e.g. Huntington's disease)^(^^20^^)^. Impaired Kyn pathway dysfunction has also been implicated in major depressive disorder. While there is much speculation over the complex mechanisms at play, immune system activation of IDO can result in an increased supply of Kyn to the central nervous system with microglial activation implicated^(^^20^^)^ in an increase in downstream Kyn metabolism and an imbalance in the ratio between neurotoxic metabolites 3-hydroxykynurenine and quinolinic acid and neuroprotective metabolite KynA^(^^20^^,^^47^^)^. Other Kyn-associated disorders include: HIV-induced dementia, Down's syndrome, Alzheimer's disease, Parkinson's disease, malignancy, multiple sclerosis and systemic lupus^(^^6^^)^. The implications of low TRP for the infant appears unclear and underinvestigated in the literature. In the first month of gestation, maternal plasma TRP is used by the fetus to synthesise serotonin, important for neurodevelopment in the forebrain^(^^18^^)^. However, disrupted organisation of the fronto-temporal lobes is one of the most noted findings in children with autistic spectrum disorder, and cells from these patients have been found to be less capable of using TRP as an energy source^(^^18^^)^. In children with attention-deficit disorder, shorter pregnancies and lower birth weights were associated with statistically increased levels of 3-hydroxykynurenine and IFN-γ^(^^48^^)^. Whether these low levels of free TRP and its metabolism in preterm EBM are reflected in infant plasma levels, and what effect if any this has on the neurological development of vulnerable exclusively breast-fed preterm infants, is an open question.

During pregnancy, while the diurnal secretion of cortisol is maintained, the regulation of the maternal hypothlamic–pituitary–adrenal axis undergoes major changes, with plasma cortisol levels rising to three times non-pregnancy levels largely as a result of corticotropin-releasing hormone (CRH) release from the placenta^(^^49^^,^^50^^)^. In the postpartum period, there is a sharp drop in placental CRH due to delivery of the placenta, followed by a slow recovery of CRH levels by 12 weeks postpartum, with cortisol levels remaining at normal levels due to corticosteroid-binding globulin and adrenal gland hypertrophy from pregnancy^(^^49^^)^. Contrary to our original hypothesis that the higher stress levels associated with preterm birth would be reflected in preterm EBM, the mothers of preterm infants did not exhibit elevated levels of stress as indicated by EBM cortisol concentrations. As cortisol triggers TDO-led breakdown of TRP down the Kyn metabolite pathway and because our results showed less free TRP in preterm EBM, it would have been reasonable to speculate that there might be higher levels of cortisol in preterm EBM. It should be noted, however, that using EBM cortisol measures as a proxy for more validated sources (e.g. plasma or saliva) may not be ideal and requires further investigation.

While increased amniotic cytokines are associated with preterm labour^(^^51^^)^, we did not find evidence of increased cytokines in preterm EBM, a pattern also demonstrated by Ustundag *et al.*^(^^52^^)^. It is important to consider maternal health, especially in relation to cytokines in the preterm group. The increased association between preterm labour, advanced maternal age (≥35 years) and higher Caesarean section rates^(^^53^^,^^54^^)^ is reflected in our results where mothers who delivered preterm had higher Caesarean section rates (75 % preterm *v.* 33 % term) and higher rates of advanced maternal age (37 years preterm *v.* 34 years term) compared with mothers who delivered term. The risk factors associated with preterm birth are numerous and many are immunologically mediated: inflammation, infection, uterine overdistention, uteroplacental ischaemia or haemorrhage, stress; maternal demographic characteristics, pregnancy history, adverse behaviours, and biological and genetic markers^(^^55^^)^. Nutritional status can have a large bearing on preterm birth; e.g. low BMI can cause decreased uterine blood flow and consumption of lower amounts of vitamins and minerals, whereas obese women are more likely to have infants with congenital abnormalities or develop pre-eclampsia and diabetes resulting in preterm labour^(^^55^^)^. The effect of various dietary components which increase plasma TRP, e.g. protein and carbohydrates^(^^56^^,^^57^^)^, are also of major significance. Interestingly, dietary supplementation with either oral l-tryptophan or α-lactalbumin (a protein high in TRP content) increases the free TRP component of breast milk but not the total TRP content^(^^33^^)^. Maternal diet thus plays an important role in TRP availability. We cannot, however, confirm that differences in maternal health and diet between term and preterm mothers underpin our results since we did not capture this information in our study.

### Limitations

It is possible that lower free TRP levels found in preterm EBM in our study could be compensated for by infants who may consume more EBM per kg body weight than their term counterparts. Infant-weighing studies have demonstrated significant variation with infants nursing anything from eight to twenty times per d while lactation is being established, and milk production by the first 2 weeks ranging from 480 to 660 ml/d^(^^58^^)^.

Measuring serum TRP levels and its metabolites in infant bloods would address this. However, this was beyond the scope of the present study. This study was limited to fat-rich hind-milk EBM investigation but does not provide any information on how TRP and its metabolites, cortisol and cytokine levels vary over the course of the lactation cycle, in particular foremilk. While we believe there is validity in our single sample collection point in that it represents a snapshot of the morning downward fluctuation of TRP in term and preterm EBM, our data support the future assessment of TRP across its diurnal cycle to see if the differences we have noted are sustained over a longer period. The same is true of diurnal variations of cortisol. EBM might not be the best biological source to use as a predictive weathervane when looking at maternal stress and immune status. Maternal plasma TRP levels were not measured. Consequently, it was not possible to determine if the TRP and metabolite concentrations present in EBM are a consequence of enzymic activity in the breast itself or are reflective of circulating maternal serum concentration. Similarly, as we did not have access to infant plasma samples, it was not possible to determine if altered TRP and Kyn metabolite supply translated into altered circulating availability in the infant. Once these valuable, difficult-to-acquire samples are harvested, it will also be important to establish the extent to which the free TRP concentration in EBM reported here contributes to circulating levels relative to the proportion derived from breast milk proteins. We did not control for the impact of maternal dietary TRP on breast milk or maternal health in our study; thus this warrants investigation in all future work. Measuring anti-inflammatory markers in future studies would also provide further valuable data on the inflammatory processes at play in breast milk. Finally, breast milk is widely recognised as being notoriously difficult to examine due to its ‘biochemical complexity’, the small concentration of many of its bioactive components, and the fact that it exhibits many dynamic and qualitative changes^(^^2^^)^, including circadian rhythms^(^^19^^,^^43^^)^. The small number of EBM samples taken at only one time point each day in this exploratory study may have impaired our ability to reliably detect group differences of these bioactive components, if they exist.

### Implications and recommendations

If the dynamics of free and total TRP are replicated and validated in larger studies, future research would be needed to establish the merits of matching full term complement of TRP and its metabolites to preterm EBM. Future research should also set out to identify the EBM metabolite profile downstream of Kyn production along the neurotoxic arm of the pathway. Clearly, if neurotoxic metabolites such as 3-hydroxykynurenine and quinolinic acid are also present in breast milk in addition to KynA, the relevance for the vulnerable infant central nervous system needs to be established. Further research would be valuable in examining the hypothesis that TRP and its metabolites present in breast milk could play a nurturing and regulating function on the immature TRP and Kyn pathways in the infant. Combining EBM cortisol analysis with maternal and infant serum, saliva samples, and validated rating scales to capture the self-reported stress status of the mothers, in conjunction with morning and evening EBM samples should be incorporated into future studies arising from this work. Before any practical conclusions and recommendations can be considered, additional studies are needed to confirm or reject our observations and hypothesis.

## Supplementary material

For supplementary material accompanying this paper visit https://doi.org/10.1017/jns.2017.69 click here to view supplementary material
