# Supplementary material for: Tryptophan metabolic profile in term and preterm breast milk: implications for health
Source: J Nutr Sci. 2018 Apr 4;7:e13. doi: 10.1017/jns.2017.69 (PMC5906556; doi:10.1017/jns.2017.69)
Supplement: Supplementary file 1 [file S2048679017000696sup001.doc]

**Supplementary Table S1**

| Biomarker | FT - Day 7 | FT - Day 14 | PT – Day 7 | PT – Day 14 |
| --- | --- | --- | --- | --- |
| TNFα | 11 | 11 | 10 | 10 |
| IL-8 | 11 | 12 | 9 | 10 |
| IL-6 | 11 | 11 | 11 | 10 |
| IL-1β | 11 | 11 | 10 | 10 |
| IFN-γ | 9 | 9 | 6 | 7 |
| Free TRP | 11 | 11 | 11 | 10 |
| Total TRP | 12 | 12 | 12 | 11 |
| Free:Total TRP | 11 | 11 | 11 | 10 |
| Kynurenine | 7 | 10 | 5 | 9 |
| Kynurenic acid | 12 | 12 | 9 | 11 |
| Kyn:FreeTRP ratio | 7 | 10 | 5 | 9 |
| Kyn:KA ratio | 7 | 11 | 5 | 9 |

n = number data points in each group, for each time point.
